# Exploring the Genetic Causes for Postnatal Growth Failure in Children Born Non-Small for Gestational Age

**DOI:** 10.3390/jcm12206508

**Published:** 2023-10-13

**Authors:** Yoo-Mi Kim, Han-Hyuk Lim, Eunhee Kim, Geena Kim, Minji Kim, Hyejin So, Byoung Kook Lee, Yoowon Kwon, Jeesu Min, Young Seok Lee

**Affiliations:** 1 Department of Pediatrics, Chungnam National University Sejong Hospital, Sejong 30099, Republic of Korea; luke2178@cnuh.co.kr (E.K.); drgnkim@naver.com (G.K.); alpineheidi@hanmail.net (M.K.); dreams_maybe@hanmail.net (H.S.); raphael@cnuh.co.kr (B.K.L.); youyisi68@gmail.com (Y.K.); jeesu.min@gmail.com (J.M.); 2 Department of Pediatrics, School of Medicine, Chungnam National University, Daejeon 34134, Republic of Korea; damus@cnu.ac.kr; 3 Department of Pediatrics, Chungnam National University Hospital, Daejeon 35015, Republic of Korea; 4 Department of Radiology, Chungnam National University Sejong Hospital, Sejong 30099, Republic of Korea; dkrad@cnuh.co.kr

**Keywords:** short stature, exome sequencing, genetic diseases, non-small for gestational age, growth hormone

## Abstract

The most common causes of short stature (SS) in children are familial short stature (FSS) and idiopathic short stature (ISS). Recently, growth plate dysfunction has been recognized as the genetic cause of FSS or ISS. The aim of this study was to investigate monogenic growth failure in patients with ISS and FSS. Targeted exome sequencing was performed in patients categorized as ISS or FSS and the subsequent response to growth hormone (GH) therapy was analyzed. We found 17 genetic causes involving 12 genes (*NPR2*, *IHH*, *BBS1*, *COL1A1*, *COL2A1*, *TRPS1*, *MASP1*, *SPRED1*, *PTPTN11*, *ADNP*, *NADSYN1*, and *CERT1*) and 2 copy number variants. A genetic cause was found in 45.5% and 35.7% of patients with FSS and ISS, respectively. The genetic yield in patients with syndromic and non-syndromic SS was 90% and 23.1%, respectively. In the 11 genetically confirmed patients, a gain in height from −2.6 to −1.3 standard deviations after 2 years of GH treatment was found. The overall diagnostic yield in this study was 41.7%. We identified several genetic causes involving paracrine signaling, the extracellular matrix, and basic intracellular processes. Identification of the causative gene may provide prognostic evidence for the use of GH therapy in non-SGA children.

## 1. Introduction

Short stature (SS) is defined as height below the third percentile for age, sex, and race, or two standard deviations (SD) below the mean [1]. Linear growth during childhood results from normal growth plate chondrogenesis regulated by nutrition, inflammatory cytokines, endocrine signals, extracellular fluid, primary properties, and molecular regulators of chondrocytes [1,2]. SS is caused by decreased growth plate chondrogenesis, which is affected by multiple molecular defects involving hormones, paracrine factors, extracellular matrix (ECM) molecules, and intracellular processes [1,2,3].

The genetic causes of growth plate disorders have been recently investigated using next-generation sequencing (NGS) [3,4,5]. Growth plate disorders caused by defects in RAS-MAPK signaling and cartilage ECM disorders, which are difficult to diagnose due to genetic polymorphisms, are potential mechanisms of idiopathic or familial short stature (FSS) characterized by postnatal growth failure [6,7,8,9,10].

Traditionally, the most common cause of SS is idiopathic short stature (ISS), including FSS, while the constitutional delay of puberty and growth (CDGP) is considered a benign condition. FSS may be an autosomal dominant genetic SS, such as skeletal dysplasia, or other conditions affecting the growth plate [11]. NGS has provided new insights into the pathogenesis and characteristics of SS [12,13], and recent studies on the application of genetic testing in ISS have been published [14,15,16]. The American College of Medical Genetics (ACMG) has also updated its algorithm for genetic testing in SS based on evidence-based practice guidelines [17].

Children born small for gestational age (SGA) without catch-up growth provide a strong indication for genetic evaluation; recombinant human growth hormone (rhGH) therapy was approved in 2001 for their treatment [18,19]. However, the cause of SS in non-SGA infants with no abnormal endocrine function is currently unclear, since they are categorized as ISS or FSS. In the case of a child with ISS where the cause of the disease is unidentified, it is difficult to further evaluate the cause of growth failure and explain the proper prognosis or milestone. Moreover, since the effect of GH treatment has not been elucidated owing to the heterogeneous characteristics of ISS or FSS, it is easy to depart from treatment. Therefore, this study aimed to investigate the genetic causes of ISS and FSS in children using NGS.

## 2. Materials and Methods

### 2.1. Patients

**Figure 1:**
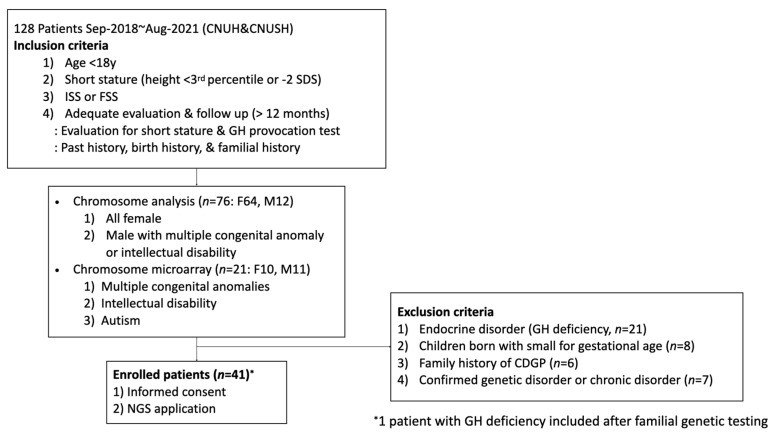
Flow chart of patient enrollment.

This study was approved by the Institutional Review Boards (IRB) of Chungnam National University (CNU) Hospital (IRB No. 2018-12-054) and CNU Sejong Hospital (IRB No. 2020-07-007). The patients were enrolled between September 2018 and August 2021 at CNU Hospital and CNU Sejong Hospital. Enrollment for the study was based on the following inclusion criteria (Figure 1): (1) age below 18 years; (2) SS defined as height less than the third percentile or −2 SD in individuals of the same sex and age by substituting the 2017 growth curve for Korean children and adolescents [20]; (3) ISS or FSS defining by maternal height <151.6 cm (−2 SD) or paternal height <163.8 cm (−2 SD) [20]; and (4) adequate evaluation and follow up (for at least 12 months). The exclusion criteria were: (1) GH deficiency or other pituitary hormonal deficiencies, such as hypothyroidism, adrenal disease, hypogonadism, and diabetes insipidus; (2) SGA without catch-up growth; (3) a parental history of CDGP; (4) children with organic brain lesions, abnormal karyotyping, other thyroid and renal diseases, congenital metabolic disease, diabetes mellitus, or a history of growth-related drug therapy; or (5) inadequate follow up. Birth weight was verified from the birth certificate from the birth institution, and patients were excluded if birth weights were below the third percentile for gestational age (GA) and sex in the Korean reference compatible with SGA [21]. Puberty was assessed according to the method described by Marshall and Tanner, and bone age was assessed according to the method described by Greulich and Pyle. Chromosome study was performed for all girls presenting with SS to exclude Turner syndrome. Chromosome microarray (CMA) was selectively performed for patients with unknown causes of developmental delay or intellectual disability, autism, or multiple congenital anomalies. All girls with Turner’s syndrome were excluded based on chromosomal studies. GH deficiency was confirmed when two different GH provocation tests showed peak GH levels <10 ng/dL. Combination with arginine and levodopa or insulin and levodopa was used for two pharmacologic stimuli and blood samples were drawn every 30 min for 2 hours. At least six hours of fasting was implemented for the GH provocation test. All patients were examined by pediatric endocrinologist. All patients were considered to exhibit ISS or FSS. Patients initiating GH treatment were assessed over a follow-up period of at least one year. Written informed consent was obtained from all participants or their parents.

### 2.2. Materials

DNA was isolated from blood samples for target exome sequencing. Analysis was performed using the Illumina MiSeq platform and TruSight One Panel (Illumina Inc., San Diego, CA, USA). Segregation analysis was performed for all cases where the variant was detected in the proband. The detected variants were classified by five variant classification categories (pathogenic, likely pathogenic, uncertain significance, likely benign, and benign) according to the ACMG guideline [22]. Each pathogenic criteria was scored as very strong (PVS1), strong (PS1–4); moderate (PM1–6), or supporting (PP1–5) [22].

Non-synonymous variants and nucleotide mutations located within 13 bases of each exon were collected, and mutations were substituted through the Genome Aggregation Database (gnomAD) and the Korean Reference Genome Database (KRGDB); allele frequencies above 1% of the general population were considered normal variants and excluded. The clinical relevance of the symptoms and detected mutations was investigated. The clinical significance of the mutations was evaluated by conducting parent and sibling tests simultaneously.

If the patients and their parents decided to receive GH therapy after the diagnosis of genetic SS, we prospectively followed the patients’ growth profile, laboratory results, and radiological findings during GH treatment. The predicted adult height (PAH) was assessed by the Greulich and Pyle method at the latest evaluation.

### 2.3. Statistical Analysis

The study population was characterized using the mean ± standard deviation (SD) and/or median for continuous descriptive variables, and categorical variables were expressed as frequencies. Descriptive and comparative statistical analyses were performed using SPSS Statistics for Windows, Version 26.0 (IBM SPSS Statistics for Windows, Version 26.0. IBM Corp, Armonk, NY, USA). Differences between groups were tested using *t*-test or Kruskal–Wallis and Fisher’s exact tests, as appropriate. Wilcoxon signed rank was used to assess the difference of height SDS in patients before and after GH treatment. Statistical significance was set at *p* < 0.05.

## 3. Results

### 3.1. Clinical Characteristics

A total of 41 patients (22 females and 19 males) from 36 unrelated families were enrolled in this study. Patients’ age ranged from 5 months to 17 years (median 6.7 years, mean 6.5 ± 4.0 years). The mean height SD score (SDS) of the patients was 2.7 ± 1.6 SDS. Their midparental height was −0.5 ± 0.9 SDS. There were 22 unrelated patients with ISS and 19 patients from 14 unrelated families had FSS. Ten patients were suspected to have syndromic SS due to cognitive issues or dysmorphic features.

### 3.2. Genetic Results

**Figure 2:**
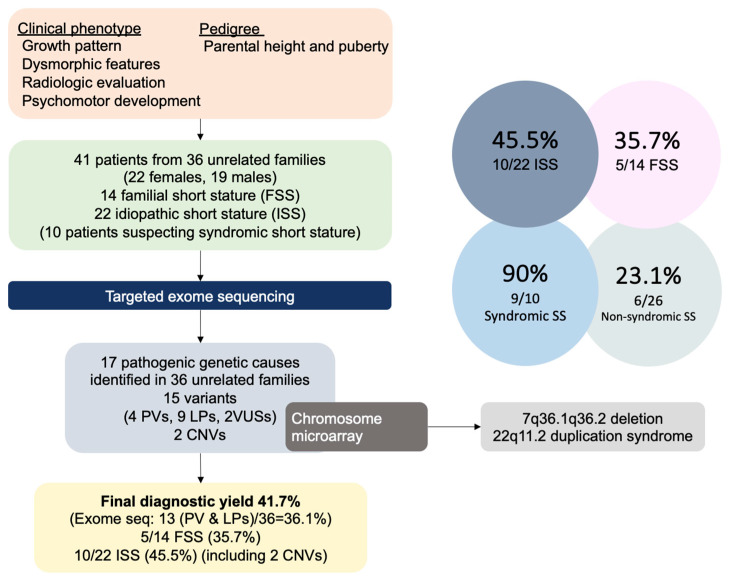
Schematic study representation of patients with short stature born non-small for gestational age.

Among the 41 patients with SS, 18 patients from 15 families had causative gene mutations, and 2 unrelated ISS patients had pathogenic copy number variants (CNVs). The total diagnostic yield for molecular changes (13 pathogenic causes in 36 families) was 36.1%. The combined positivity rate for pathogenic molecular changes and CNVs was 41.7% (Figure 2).

In 19 of the 14 unrelated patients with FSS, genetic causes of SS were found in 7 families, involving *NPR2*, *COL1A1*, *ADNP*, *TRPS1*, *SPRED1*, and *PTPTN11*, of which 4 variants were novel. Of the 22 patients with ISS, 10 were found to have genetic causes. The causative genes were *BBS1*, *IHH*, *COL1A1*, *COL2A1*, *MASP1*, *NADSYN1*, and *CERT1*. Seven novel variants were identified in this study. Interestingly, pathogenic CNVs were detected in two patients with ISS following a preliminary indication using exome sequencing.

A 13-month-old boy (participant 3) with suspected Noonan syndrome (NS) was diagnosed with 22q11.2 microduplication syndrome (Table 1). Initial karyotyping revealed a balanced translocation of 46, XY, t(12;14) (q15;q22). Exome sequencing revealed no pathogenic mutations, but a suspected duplication at 22q11.21. CMA confirmed a 22q11.21 duplication of 2.5 Mb [GRCh37/hg19 22q11.21(chr22:8,916,842-21,465,659) × 3]. In an 11-year-old girl (participant 30) with normal karyotype, a deletion at 7q36 was suspected by exome sequencing (Table 1). Additional CMA revealed a 4 Mb deletion at 7q36.1q36.2 [GRCh37/hg19 7q36.1q36.2 (chr7:150,796,099-155,074,825) × 1].

In this study, a genetic cause was identified in nine out of ten patients suspected with syndromic SS (90%) (Table 1, Figure 2).

### 3.3. Paracrine Signaling Defects

Four causative genes, *NPR2*, *IHH*, *BBS1*, and *TRPS1*, were identified and found to be related to paracrine signaling (Table 2).

**Figure 3:**
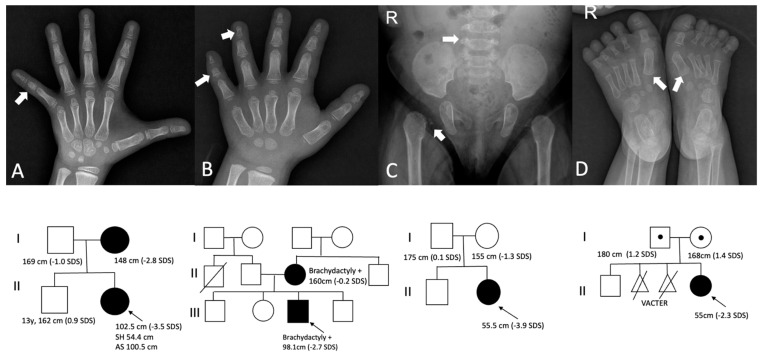
Radiological findings and pedigree in patients with *NPR2* (participant 16), *IHH* (participant 12), *COL2A1* (participant 2), and *NADSYN1* gene (participant 1) variants (**A**–**D**). Simple X-ray of the hand showing a shortened and dysplastic middle phalanx in participant 16 with the *NPR2* variant (**A**) and brachydactyly and shortening of phalanxes in participant 12 with the IHH variant (**B**). Pelvic X-ray in participant 2 showing the small iliac bone and small irregular epiphyses of both proximal femurs with widening and metaphysis irregularities of both femoral heads and short neck. The white arrow shows the relatively smaller size of the L5 body compared to the L4 body (**C**). Varus deformity of first metatarsal bone in participant 1 (**D**).

The *NPR2* variant was found in participant 16 (height −3.5 SDS) and her affected mother (height −2.8 SDS) (Table 2). The *NPR2* (NM_003995.4) variant was c.1099A>G (p.Lys367Glu). Both the patient and mother showed fifth clinodactyly, and radiological findings revealed a shortened and dysplastic middle phalanx in the fifth finger (Figure 3A).

*IHH* (NM_002181.4) variant of c.683G>T (p.Arg228Leu) was detected in participant 12 having a proportionate SS, brachydactyly, and fifth clinodactyly. Radiography revealed shortened and dysplastic middle and distal phalanxes (Figure 3B). This variant was inherited from the mother, who also had brachydactyly despite having a normal height (−0.2 SDS).

Participant 36 with syndromic obesity (BMI 34.5 kg/m^2^) and SS had compound heterozygous *BBS1* (NM_024649.4) mutations (Table 2). The patient presented with tapered fingers and brachydactyly. After diagnosis, retinal dystrophy, including foveal depigmentation and macular thinning, renal cysts, multiple hepatic hemangiomas, and a functional ovarian cyst sized 2.3 cm were identified from systematic surveillance.

Participant 31 displayed SS, sparse hair and eyebrows, and a pear-like nose. In addition, cone-shaped phalanxes were identified on hand radiography. Exome sequencing identified the *TRPS1* (NM_014112.5) mutation c.2686dup (p.Gln896Profs*8) in this participant and the mutation was inherited from the father (height −2.7 SDS).

### 3.4. Extracellular Matrix Defects

*COL1A1*, *COL2A1*, and *MASP1* were identified in this study (Table 2). Two unrelated patients manifested *COL1A1* mutations. Participant 18 inherited the *COL1A1* (NM_000088.4) variant of c.3766G>A (p.Ala1256Thr) from her mother (height 149 cm, −2.6 SDS). This variant was previously reported in a 41-year-old male with type 1 osteogenesis imperfecta [23]. Participant 8 experienced long bone fractures twice during infancy prior to trauma. Exome sequencing revealed a novel *COL1A1* (NM_000088.4) mutation, c.757C>T (p.Arg253*). His father and grandfather carried this mutation and displayed different heights, of 176 cm (0.3 SDS) and 160 cm (−2.7 SDS), respectively. All patients displayed elbow dislocation, blue sclera, hearing defects, and recurrent fracture history during childhood.

Two patients (participants 2 and 20) exhibited a *COL2A1*-related disorder (Table 2). The radiological findings of participant 20 were compatible with those of spondyloepiphyseal dysplasia (Figure 3C).

Participant 13 with profound SS (height −3.1 SDS) had compound heterozygous mutations with c.851C>T (p.Ser284Leu) and c.744 + 7C>T (splicing) in *MASP1* (NM_139125.3). She experienced feeding intolerance and recurrent upper respiratory tract infections. No definite developmental delays were observed.

### 3.5. Fundamental Cellular Process

*SPRED1*, *PTPN11*, *ADNP*, *NADSYN1*, and *CERT1* variants were identified in this study (Table 2).

Participant 7 had a c.950C>G (p.Ser317*) in the *SPRED1* (NM_152594.2) gene, which was inherited from the father. The father displayed a thick nasolabial fold, macrocephaly, and a triangular face. This participant showed macrocephaly (2.8 SDS) and dysmorphic features (Table 1). Fine motor skills only were delayed until 33 months of age for all milestones.

Two families with FSS were diagnosed with NS. Participant 24-1 exhibited FSS; his brother (participant 24-2) and younger sister (participant 24-3) also showed postnatal growth failure. Exome sequencing revealed a known mutation in *PTPN11* (NM_002834.5), c.209A>G (p.Lys70Arg), inherited from the mother (−1.5 SDS, Table 1). These siblings showed clinical variability in intellectual ability and height within their families (Table 1). No abnormal echocardiographic findings were observed in these siblings.

Participant 4 had typical facial features and feeding intolerance, but no developmental delay (Table 1). A novel *PTPN11* variant, c.1511T>C (p.Met504Thr) inherited from the father (height −0.1 SDS) was identified. Her father was regularly followed up for arrhythmia and pectus carinatum, but no structural anomalies were observed on echocardiography.

Participant 32-1 displayed an *ADNP* (NM_015339.5) variant for c.2992G>T (p.Asp998Tyr), which was inherited from the non-SS mother. Further familial testing was performed on two older sisters; one older sister (participant 32-2) with the *ADNP* variant had idiopathic GH deficiency (GH peak 0.9 ng/mL and 6.7 ng/mL) and early puberty. The sisters with the *ADNP* variant had a prominent forehead with a high anterior hairline, a down-slanting palpebral fissure, a low nasal bridge, a bulbous nose, and a pointed chin similar to their mother.

Participant 1 was born extremely prematurely owing to the premature rupture of membranes at labor. The participant also had a shortened limb, a prominent forehead, bulbous nose, and a low nasal bridge. At 5 months of age, her growth velocity was stunted (Table 1). The CMA was normal, but two compound heterozygous mutations, c.164A>T (p.Asp55Val) and c. 1048-1G>T (splicing) in *NADSYN1* (NM_018161.5) were identified (Table 2). This participant showed only gross motor delay at 12 months of age and speech delay at 2 years of age. A skeletal survey revealed coxa valgum and varus deformity of the first metatarsal bone (Figure 3D). The patient had severe feeding intolerance and sleep disturbances, and hypercalcemia due to vitamin D intoxification was noted at 15 months of age. Her calcium level increased to 12.9 mg/dL with a suppressed parathyroid hormone level (0.2 pg/mL). The 25-OH vitamin D_2_ concentration and the urinary calcium-to-creatinine ratio increased to 105.9 ng/mL and 2.5, respectively. Renal ultrasonography revealed nephrocalcinosis. All medications, including a high dose of cholecalciferol (800 IU/d) from diverse self-nutrients, were discontinued, and the Locasol^®^ formula (Nutricia Corp., Hoofddorp, The Netherlands) was started for hypercalcemia. Since then, the calcium level has been maintained at 9.0–10.1 mg/dL. This participant started GH treatment at 30 months of age and growth velocity was accelerated to 3.4 cm per 3 months of treatment.

Participant 35 exhibited profound SS and severe intellectual disabilities. The CMA and genetic testing for *MECP2* and Angelman syndrome returned normal results. Exome sequencing revealed a de novo *CERT1* (NM:001130105.1) variant for c.779C>T (p.Ser260Leu) resulting in intellectual developmental disorder, autosomal dominant 34 (MIM# 616351).

### 3.6. Growth Response to rhGH Treatment

**Figure 4:**
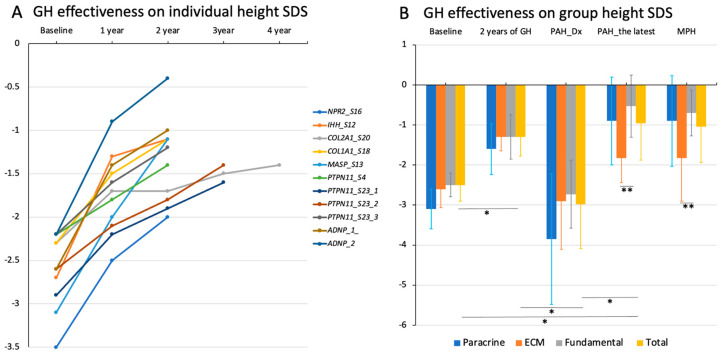
Height gains after recombinant human growth hormone treatment by (**A**) individual and (**B**) group categories (paracrine signal defect, extracellular matrix (ECM) defect, fundamental cellular defect, and total group) * height SDS was significantly increased at 2 years of GH treatment, and predicted adult height (PAH) at the latest evaluation in fundamental group and total group (* *p* <0.05); ** the MPH and PAH at latest evaluation between ECM defect and fundamental cellular defect group showed significant difference (** *p* < 0.05).

Of the 15 patients with a genetic variant, 11 patients received rhGH for 2.8 ± 0.6 years (range, 2–3 years 10 months) (Figure 4A). Participant 1 with NADSYN1 variants was excluded from the analysis due to the short period of GH treatment. The median age at GH treatment was 7 years of age (range, 2–11 years), and all patients height significantly increased to −1.3 ± 0.5 SDS at 2 years after GH treatment compared to baseline height −2.6 ± 0.4 SDS (Figure 4B, *p* = 0.003). When we calculated their PAH at diagnosis and the latest evaluation, their PAH at the latest evaluation was also significantly increased to −1.0 ± 0.9 SDS compared to the PAH SDS at diagnosis (−3.0 ± 1.1) (*p* = 0.003) (Figure 4B).

Regarding paracrine signaling defect, two patients (*NPR2* and *IHH* variant) were treated with GH therapy. Participant 16 with the *NPR2* variant showed a height gain from −3.5 SDS to −1.6 SDS after three years of GH treatment (Figure 4A). As advanced bone age (10 years) and breast budding were noted at the age of 8 years and 6 months, a gonadotropin-releasing hormone (GnRH) stimulation test was performed. The results showed a prepubertal pattern (LH peak 2.7 mIU/mL). The subject showed transient precocious puberty, and pubertal progression was followed up regularly. The height SDS of participant 12 improved by −1.1 SDS after two years of GH treatment (Figure 4A).

In the ECM defect group, three patients (*COL1A1*, *COL2A1*, and *MASP1*) received rhGH. Their height and PAH SDS at baseline, height SDS after 2 years of rhGH, and PAH at the latest evaluation were −2.6 ± 0.5 SDS, −2.9 ± 1.5 SDS, −1.3 ± 0.3, and −1.8 ± 0.6 SDS, respectively. As participant 18 was diagnosed with central precocious puberty (CPP) at the age of 7 years, combined treatment with a GnRH analog and GH therapy was subsequently started. During treatment, her height increased from −2.3 SDS to −1.1 SDS over two years. Participant 13 with *MASP* variants showed a height gain to −1.5 SDS after two years of GH treatment, but combined treatment with GnRH analog was required due to the diagnosis of CPP at the age of 7 years and 8 months. Participant 20 with the *COL2A1* variant received GH treatment for three years and ten months. Although his height increased from −2.3 SDS to −1.4 SDS after GH treatment for about four years, the PAH was reduced to −2.5 SDS for his advanced BA.

In the fundamental cellular defect group, a total of six patients (*PTPN11* and *ADNP* variant) were treated with the rhGH (Figure 4A). Their initial height SDS was increased from −2.5 ± 0.3 SDS to −1.3 ± 0.6 SDS, and the initial PAH SDS (−2.7 ± 0.9) was also significantly improved by −0.5 ± 0.8 PAH SDS at the latest evaluation (Figure 4B).

In addition, there were no significant differences in height SDS between the group at baseline and 2 years after rhGH. However, MPH and PAH SDS at the latest evaluation was significantly higher in the fundamental cellular defect group than in the ECM group (Figure 4B, *p* < 0.05).

## 4. Discussion

The results of this study demonstrated the importance of genetic analysis in patients with ISS and FSS. The genetic defects identified in this study involved various molecular pathways, including paracrine signaling, ECM molecules, and fundamental intracellular processes, highlighting the complex nature of growth regulation.

Furthermore, genetic yields differed between patients with syndromic and non-syndromic SS. Ten patients (four FSS, six ISS) were suspected of syndromic SS, among which nine were attributed to a genetic cause (90%); however, the genetic yield of non-syndromic SS patients was 23.1% (6/26). Patients with syndromic SS exhibited a higher rate of genetic identification than patients with non-syndromic SS, emphasizing the need for genetic evaluation in these cases. In contrast, the diagnostic yield in patients with non-syndromic SS was relatively low, suggesting that additional factors beyond genetic causes may contribute to growth impairment.

Interestingly, families with the same gene mutations (*IHH*, *COL1A1*, *SPRED1*, *PTPN11*, and *ADNP*) exhibited height variability. The penetrance of *ADNP* mutations was less than 100%; patients in two families have been reported with ADNP-related disorders wherein the pathogenic variant has been inherited from an unaffected parent [24].

Natriuretic peptide receptor 2 (NPR2) is a receptor for the C-type natriuretic peptide, which stimulates growth plate chondrogenesis [25,26]. In particular, *NPR2* heterozygous mutations reportedly have genetic causes in 2–6% of patients with ISS [27,28,29]. In addition, these patients tend to have typical findings, including brachydactyly, shortened metacarpal bone, and clinodactyly. GH treatment significantly improved the height SDS of patients with *NPR2* heterozygous mutations [30,31]. Despite the small sample size in our study, *NPR2* mutations accounted for 2.9% of the ISS or FSS cases, and this patient exhibited a height gain of 15 cm during the two years of GH treatment.

We further identified a male child with ISS harboring an *IHH* variant and demonstrating brachydactyly, shortening, and a dysplastic middle phalanx of the fifth finger. In addition to *ACAN* mutation, *IHH* is a common mutation in ISS with mild skeletal anomalies [32]. A high prevalence (81%) of *IHH* and *ACAN* mutations has been reported among 21 SS patients identified as having genetic causes of ISS [33]. These patients exhibited typical radiological findings, including middle phalanx shortening of the fifth finger. The response to rhGH has been encouraging, with a mean height gain of 0.6 SDS after one year of GH treatment [33]. Our patient was also a good responder, demonstrating +1.7 SDS height change in two years of GH treatment.

Transcriptional repressor GATA binding 1 (TRPS1) has multiple functions for chondrocyte differentiation and mineralization by interacting with IHH/GLI3 [34,35].

*MASP1* on chromosome 3q24 encodes for three isoforms, mannan-binding lectin serine peptidase 1 (MASP1), MASP3, and MASP4, and is a disease-causing a gremlin mutation, resulting in 3MC syndrome (MIM#600521) [36]. The 3MC syndrome is a rare autosomal recessive disorder characterized by craniofacial dysmorphisms, including highly arched eyebrows, ptosis, blepharophimosis, cleft lip/palate, postnatal growth failure, hearing loss, and varying degrees of cognitive impairment [36]. To date, 30 patients from 22 families presenting with 3MC syndrome 1 caused by *MASP1* mutations have been reported in the literature [36,37,38,39]. Biallelic *MASP1* mutations have been identified in girls with ISS exhibiting normal intelligence, mild facial features, and arched eyebrows. Two novel mutations (c.744 + 7C>T and c.851C>T) in *MASP1* (NM_139125.3) in the donor splice site affecting exons 5 and 6 and were shared by three isoforms. They are predicted to affect the CUB2 domain, the second most common domain, followed by the serine protease domains, in which mutations causing 3MC syndrome have been frequently reported [37,38,39]. Although the mechanism of postnatal growth failure in 3MC syndrome is unclear, MASP1 has been indicated to play a role in extracellular biological processes, and its molecular function is related to calcium ion binding and ECM components [40].

ADNP-related disorders are genetic causes of autism spectrum disorder, presenting with mild to moderate intellectual disability; developmental delay; multiple anomalies including heart, urogenital, and skeletal defects; and facial features including prominent and high forehead, low nasal bridge, down-slanting palpebral fissures, small chin, and prominent eyelashes [24]. Interestingly, all reported cases have truncated mutations, including nonsense or frameshift mutations, and almost all patients (97.1%) harbor de novo *ADNP* mutations [37]. Approximately 25% of individuals exhibit SS or GH deficiencies [37]. Since C-terminal ANDP mutations in the two patients were inherited from unaffected parents, the penetrance of ADNP was not considered to be 100% [24]. Our patient was familial and showed no developmental issues, exhibiting only facial features and SS. Notably, the patient carried missense variants located at the C-terminus, which, to our knowledge, have not previously been reported. Considering the limited information on missense ADNP variants, further investigations are needed to establish genotype–phenotype correlations.

The *CERT1* gene, termed as *COL4A3BP*, was identified in three patients with neurodevelopmental disorders, possessing a p.Ser132Leu mutation in *CERT1* [41]. Our patient had p.Ser260Leu, and a serine-to-leucine substitution in the serine-repeat motif was considered to impair protein function [41,42].

In this study, we identified a patient with *NADSYN1*-associated nicotinamide adenine dinucleotide (NAD) deficiency disorders demonstrating severe postnatal growth failure, and mild skeletal anomalies without global development. The first report of *NADSYN1*-associated NAD deficiency disorder specifies multiple severe anomalies and no living patients [43]. However, high clinical variability in *NADSYN1*-associated NAD deficiency disorders has recently been reported [44,45]. The clinical phenotype of *NADSYN1*-associated NAD deficiency is similar to that of the VATER syndrome. Our patient had a family history of abortions due to the VATER syndrome; however, genetic testing for *NADSYN1* was not available.

Two patients had CNVs involving chromosome 22q11.2 or 7q36, as determined using NGS. The duplicated region at 22q11.21 included genes, such as *LZTR1*, *TBX1*, *SERPIND1*, and *TUBA8*, among which *LZTR1* is a known causative gene of NS 10 (MIM#616564). Gain of function for *LZTR1* is responsible for NS 10 [46]. Considering previous reports regarding the duplication of *PTPN11* and *RAF1* [47,48,49,50]; further evaluation is required to correlate 22q11.2 microduplication syndrome encompassing *LZTR1* with NS 10. A 7q36.1q36.2 deletion containing several pathogenic genes, including *DPP6*, *KMT2C*, *ASB10*, *PRKAG2*, *KCNH2*, among which *KMT2C* is a causative gene for Kleefstra syndrome 2 (MIM#617768). This syndrome is characterized by variable intellectual disability, mild dysmorphic features, SS, and brachycephaly [51].

Our study demonstrated the high diagnostic yield (47.2%) in non-SGA SS patients compared to the general detection rate of 27.1% (95% CI, 18.1–37.2%), reported in a meta-analysis of 20 studies of patients with SS [52].

In comparison to the SGA SS group, defect of GH-IGF axis or imprinting disorder is main cause for SGA without catch-up growth in addition to the growth plate dysfunction [53,54]. The genes for growth plate dysfunction have been identified in ISS or FSS group (Table 3). Collagen gene mutations were detected in FSS patients (10/87, 11.5%) and of unknown origin in SS patients (18/232, 7.8%) [55,56].

Importantly, our study highlighted the effectiveness of GH therapy in patients with growth failure. Although the identified genetic defects involving the ECM may limit the response to GH treatment, most patients in this study displayed height gain after GH treatment. There have been several efforts to assess the efficacy of GH treatment on the genotype of growth plate dysfunction, despite the small numbers of patients [30,33,55,56,57]. One study of nine patients with a collagen gene defect showed a change in height SDS of 1.0 for 2.8 years of GH treatment [55]. Among them, two patients were diagnosed with CPP, and their PAH was not shown. Another study [56] showed the growth response to the rhGH in 10 FSS patients with collagen gene defects. They showed the height SDS change 0.9 after 3 years of rhGH and four patients who reached adult height showed variable height gains, ranging from –0.67 to +1.0 SD and –0.45 to +0.5 SD compared to their baseline height and the height of their affected untreated parent, respectively [56]. Our study also showed an improvement in height SDS from −2.6 to −1.3 (height SDS change 1.3) for 2.8 years of GH treatment in the ECM group. However, since all patients in the ECM group showed advanced BA during the follow-up period, the PAH at the latest evaluation was decrease to the −1.8 SDS.

These findings support the use of GH therapy in non-SGA children with SS, particularly when the causative gene is identified. Further, categorizing patients based on specific molecular defects is essential for customized treatment strategies. Understanding the underlying genetic causes can provide valuable prognostic information and aid in decision-making regarding the administration of GH therapy in non-SGA children with SS. As our study has some limitations, including a small number of patients, a short follow-up period for the efficacy of GH treatment, and the use of targeted panels instead of whole exome sequencing or whole genome sequencing, further study with a larger cohort, long-term follow-up, and comprehensive genetic study is required to evaluate the genetic aspects and rhGH effects of growth plate dysfunction in patients with ISS or FSS.

The efficiency of NGS was evaluated in these patients by identifying the cause of SS, which was initially classified as ISS or FSS. This may be helpful to implement early intervention and predict the efficacy of GH treatment using factor analysis.

## 5. Conclusions

In conclusion, our study demonstrated a diagnostic yield of 41.7% using targeted exome sequencing and identified multiple genetic causes of ISS or FSS, further elucidating the molecular mechanisms involved in growth regulation. The GH treatment was generally effective at increasing height SDS in this study, and more in the fundamental cellular defect group than the ECM defect group, despite the limited number of patients and treatment period. These findings have implications for the management and treatment of patients with ISS or FSS. Further research is warranted to explore additional genetic factors and their impact on growth outcomes.

## Figures and Tables

**Table 1:** Clinical findings for short stature children born non-small for gestational age.

No	Sex	Age	GA Week	BW kg	Mode of Delivery	Ht cm (SDS)	Wt kg (SDS)	Pat. Ht cm (SDS)	Mat. Ht cm (SDS)	MPH cm (SDS)	Psychomotor Issue	Dysmorphism	Category
1	F	5 m CA 3 m	29 + 2	1.15	C/sec	55 (−2.3)	4.9 (−1.4)	180 (1.2)	168 (1.4)	167.5 (1.3)	Gross motor delay	Prominent forehead, bulbous nose, low nasal bridge, short limb	ISS, R/O SD
2	F	5 m	37 + 5	3.16	NSVD	55.5 (−3.9)	6.0 (−1.1)	175 (0.1)	155 (−1.3)	156 (−1.0)	Gross motor delay	Prominent forehead, brachydactyly, short limb	ISS, R/O SD
3	M	13 m	40	3.51	C/sec	72.2 (−2.0)	9.1 (−0.7)	170 (−0.8)	155 (−1.3)	169 (−1.0)	None (4.6 y: IQ 120)	Macrocephaly, s/p VSD closure, resemble facial features with NS	ISS, R/O SD
4	F	15 m	35 + 5	2.57	C/sec (twin)	70.0 (−2.7)	7.9 (−1.6)	174 (−0.1)	158 (−0.6)	159.5 (−0.3)	None	FTT, sparse hair & eyebrows, long philtrum, down-slanting palpebral fissure, high forehead	ISS, R/O SD
5	M	31 m	38	2.9	C/sec	82.5 (−2.9)	10.3 (−2.3)	167 (−1.4)	161 (0.0)	170.5 (−0.7)	None	-	ISS
6	M	3 y 10 m	38	3.1	C/sec	91.6 (−2.6)	13.1 (−1.7)	178 (0.6)	153 (−1.7)	172 (−0.4)	None	-	ISS
7	F	3 y 11 m	40 + 6	3.5	C/sec	92.6 (−2.1)	12.8 (−2.0)	166 (−1.6)	148 (−2.8)	150.5 (−2.2)	None	Triangular face, prominent forehead, macrocephaly	FSS, R/O SD
8	M	4 y 4 m	40	3.6	NSVD	96.5 (−2.0)	14.6 (−1.7)	176 (0.3)	153 (−1.7)	171 (−0.6)	None	Blue sclerae	ISS
9	M	4 y 10 m	40	3	NSVD	96.7 (−2.7)	14.3 (−2.4)	169 (−1.0)	153 (−1.7)	167.5 (−1.3)	None	-	ISS
10	F	4 y 11 m	38 + 5	2.9	NSVD	98.2 (−2.3)	13.2 (−2.9)	160 (−2.7)	165 (0.8)	156 (−1.0)	None	-	FSS
11	F	4 y 11 m	40 + 6	4	NSVD	97.9 (−2.4)	15.4 (−1.4)	168 (−1.2)	155 (−1.3)	155 (−1.3)	None	-	ISS
12	M	5 y	40	3.1	NSVD	98.1 (−2.7)	18.1 (−0.4)	176 (0.3)	160 (−0.2)	175 (0.1)	None	Brachydactyly	ISS
13	F	5 y 2 m	38	2.9	C/sec	96.9 (−3.1)	14.2 (−3.7)	164 (−1.9)	158 (−0.6)	155 (−1.3)	None	-	ISS
14	M	6 y 4 m	35 + 4	2.1	NSVD	104.6 (−2.8)	18.2 (−1.5)	170 (−0.8)	160 (−0.2)	171.5 (−0.5)	None	-	ISS
15	M	6 y 5 m	39 + 5	3.9	C/sec	107.7 (−2.2)	18.5 (−1.4)	167 (−1.4)	153 (−1.7)	166.5 (−1.5)	None	-	ISS
16	F	6 y 6 m	37 + 2	2.6	C/sec	102.5 (−3.5)	16.7 (−2.1)	169 (−1.0)	148 (−2.8)	152 (−1.9)	None	Triangular face, down-slanting palpebral fissure, prominent philtrum, relative macrocephaly	FSS, R/O SD
17-1	M	6 y 8 m	40	3	C/sec	109.8 (−2.1)	17.7 (−2.1)	161 (−2.5)	148 (−2.8)	161 (−2.5)	None	-	FSS
17-2	F	7 y 7 m	40	3.03	C/sec	108.9 (−3.3)	20.8 (−1.2)	161 (−2.5)	148 (−2.8)	149 (−2.6)	None	-	FSS
18	F	6 y 11 m	38	2.7	C/sec	107.8 (−2.7)	20.2 (−0.9)	165 (−1.8)	149 (−2.6)	150.5 (−2.2)	None	Blue sclerae, triangular face, delayed eruption	FSS
19	M	7 y	39	2.8	NSVD (IVF)	105.5 (−3.6)	18.0 (−2.3)	170 (−0.8)	158 (−0.6)	170.5 (−0.7)	None	-	ISS
20	M	8 y 6 m	39 + 5	3.2	NSVD	118.1 (−2.5)	25.8 (−0.8)	153 (−4.1)	153 (−1.7)	159.5 (−2.8)	None	-	FSS
21	F	8 y 2 m	40	2.8	NSVD	111.1 (−3.5)	17.7 (−2.9)	170 (−0.8)	148 (−2.8)	152.5 (−1.8)	None	-	FSS
22	M	8 y 10 m	39 + 1	2.8	NSVD	113.0 (−3.8)	22.0 (−2.0)	170 (−0.8)	160 (−0.2)	171.5 (−0.5)	None	-	ISS
23	F	8 y 9 m	37	2.32	C/sec (Twin)	118.0 (−2.5)	21.0 (−2.1)	176 (0.3)	150 (−2.4)	156.5 (−0.9)	None	-	FSS
24-1	M	8 y 8 m	37 + 3	2.6	NSVD	119.2 (−2.3)	19.7 (−2.7)	176 (0.3)	154 (−1.5)	171.5 (−0.5)	Mild ID (IQ 64)	-	FSS
24-2	M	10 y 8 m	36 + 6	2.9	NSVD	126.8 (−2.7)	25.8 (−2.0)	176 (0.3)	154 (−1.5)	171.5 (−0.5)	None (IQ 94)	-	FSS
24-3	F	7 y 10 m	39 + 5	3.1	NSVD	117.3 (−1.6)*	16.6 (−3.2)	176 (0.3)	154 (−1.5)	158.5 (−0.5)	None (IQ 82)	-	FSS
25	F	10 y	40	3	NSVD	126 (−2.3)	32.8 (−0.3)	173 (−0.3)	160 (−0.2)	160 (−0.2)	None	-	ISS
26	M	10 y 10 m	40	3.2	NSVD	128.4 (−2.5)	23.0 (−2.7)	165 (−1.8)	149 (−2.6)	163.5 (−2.0)	Speech delay	Triangular face, narrow palpebral fissure, low nasal bridge	FSS, R/O SD
27	M	10 y 11 m	40	3.2	NSVD	126.7 (−2.9)	25.0 (−2.3)	164 (−1.9)	154 (−1.5)	165.5 (−1.7)	None	-	ISS
28	M	10 y 11 m	37 + 6	2.86	NSVD	130.1 (−2.3)	23.4 (−2.7)	164 (−1.9)	154 (−1.5)	165.5 (−1.7)	None	-	ISS
29	M	11 y 8 m	41	3	NSVD	126.5 (−3.4)	23.8 (−2.9)	170 (−0.8)	160 (−0.2)	171.5 (−0.5)	None	-	ISS
30	F	11 y	40	2.9	NSVD	122.2 (−3.7)	22.5 (−3.0)	170 (−0.8)	160 (−0.2)	158.5 (−0.5)	ADHD	s/p PDA ligation, ASD closure	ISS
31	M	11 y 11 m	38 + 2	3.3	C/sec	135.8 (−2.1)	37.3 (−0.9)	160 (−2.7)	164 (0.6)	168.5 (−1.1)	ADHD, mild ID (IQ 69)	Sparse hair and eyebrows, pear like nose	FSS, R/O SD
32-1	F	6 y	40	3.6	C/sec	103.8 (−2.6)	15.4 (−2.5)	161 (−2.5)	162 (0.2)	155 (−1.3)	None		FSS
32-2	F	10 y 1 m	40	2.8	C/sec	122.9 (−2.9)	26.8 (−1.5)	161 (−2.5)	162 (0.2)	155 (−1.3)	None		FSS (GHD)
32-3	F	11 y 10 m	38	3.3	C/sec	135.4 (−2.3)	36.6 (−0.8)	161 (−2.5)	162 (0.2)	155 (−1.3)	None		FSS
33	F	12 y	40	3.1	NSVD	134.2 (−2.6)	49.6 (0.7)	170 (−0.8)	150 (−2.4)	156 (−1.0)	None	-	FSS
34	F	12 y 6 m	37	2.64	NSVD	134 (−3.1)	27.3 (−2.9)	171 (−0.6)	151 (−2.1)	154.5 (−1.4)	None	-	FSS
35	F	14 y	36	2.2	C/sec	129.3 (−4.9)	21.2 (−6.1)	173 (−0.3)	163 (0.4)	161.5 (0.1)	Severe ID, wide gait, stereotypic behavior	Microcephaly, widely spaced teeth, hypertelorism	ISS, R/O SD
36	F	17 y	41	3.99	C/sec	151.5 (−2.0)	79.1 (2.7)	166 (−1.5)	161 (0.0)	158 (−0.6)	Moderate ID	s/p TOF, polydactyly, and ectopic anus, obesity	ISS, R/O SD

GA: gestational age, BW: birth weight, HT: height, SD: standard deviation, HT: paternal height, mat. HT, maternal height; MPH, mid-parental height; CA, corrected age; NSVD; normal spontaneous vaginal delivery, C/sec; cesarean section; ADHD, attention-deficit hyperactivity disorder; ID, intellectual disability; VSD, ventricular septal defect; NS, Noonan syndrome; PDA; patent ductus arteriosus; ASD; atrial septal defect; TOF; tetralogy of Fallot, ISS, idiopathic short stature; FSS, familial short stature; GHD, growth hormone deficiency; SD, syndromic disorder. * The height SDS of participant 24-3 was −2.1 SDS at 9 years of age.

**Table 2:** Genetic results for children born non-small for gestational age.

Pt. No.	Category	Disease	Gene	cDNA Variant	Protein Variant	MAF ^a^	In Silico Analysis ^b^	Inheritance	ACMG Classification [22]
	Paracrine signaling								
16	FSS	Short stature with nonspecific skeletal abnormalities	*NPR2*	**c.1099A>G**	**p.Lys367Glu**	0/0	D/T/D/	Mat	LP (PM1, PM2, PP1, PP4)
12	ISS	Brachydactyly, type A1	*IHH*	**c.683G>T**	**p.Arg228Leu**	0/0	T/D/D	Mat	VUS (PM2, PP3, PP4)
36	ISS	Bardet–Biedl syndrome, type 1	*BBS1*	c.1285C>T c.1339G>A	p.Arg429 * p.Ala447Thr	0.0008/0 0.00037/0	- D/T/D	Pat Mat	PV (PVS1, PM2, PM3, PP3, PP4) LP (PM1, PM2, PM3, PP3, PP4)
31	FSS	Trichorhinophalangeal syndrome 1	*TRPS1*	**c.2686dup**	p.Gln896Profs *8	0/0	-	Pat	PV (PVS1, PM2, PP1, PP4)
	Extracellular matrix (ECM) defects								
18	FSS	Osteogenesis imperfecta	*COL1A1*	c.3766G>A	p.Ala1256Thr	0.00134 /0.00045	D/D/D	Mat	VUS (PM2, PP1, PP3, PP4)
8	ISS	Osteogenesis imperfecta	*COL1A1*	**c.757C>T**	p.Arg253 *	0/0	-	Pat	PV (PVS1, PM2, PP1, PP3, PP4, PP5)
20	ISS	Type II collagen disorder	*COL2A1*	**c.889G>A**	**p.Gly297Ser**	0/0	D/T/ D	De novo	LP (PM1, PM2, PM6, PP3, PP4)
2	ISS	Type II collagen disorder	*COL2A1*	**c.3310G>C**	**p.Gly1104Arg**	0/0	D/T/D	De novo	LP (PS2, PM1, PM2, PP3, PP5)
13	ISS	3MC syndrome 1	*MASP1*	**c.851C>T** c.744 + 7C>T	**p.Ser284Leu** splicing	0/0 0.0000041/0.00045	D/D/D D/D/D	Pat Mat	LP (PM3, PM2, PP2, PP3, PP4)
	Fundamental cellular process								
24-1 24-2 24-3	FSS	Noonan syndrome	*PTPN11*	c.209A>G	p.Lys70Arg	0/0	D/D/D	Mat	LP (PM1, PM2 PP1 PP3 PP4 PP5)
4	FSS	Noonan syndrome	*PTPN11*	**c.1511T>C**	**p.Met504Thr**	0/0	D/D/D	Pat	LP (PM1, PM2, PP1, PP3, PP4)
7	FSS	Legius syndrome	*SPRED1*	**c.950C>G**	p.Ser317 *	0/0	-	Pat	PV (PVS1 PM2 PP1 PP3 PP4)
32-1 32-2	FSS	Helsmoortel-van der Aa syndrome	*ADNP*	**c.2992G>T**	**p.Asp998Tyr**	0/0	D/D/D	Mat	LP (PM1, PM2, PP1, PP4)
35	ISS	Intellectual developmental disorder, autosomal dominant 34	*CERT1* *(COL4A3BP)*	c.779C>T	p.Ser260Leu	0/0	D/D/D	De novo	PV (PS1 PS2 PM1 PM2 PP4)
1	ISS	NAD synthetase deficiency disorder	*NADSYN1*	c.164A>T **c.1048-1G>T**	p.Asp55Val **splicing**	0.00081/0 0/0	D/D/D	Pat Mat	LP (PM3, PM2, PP2, PP3, PP4)
3	ISS	22q11.2 microduplication syndrome (Noonan syndrome 10)	*LZTR1*	duplication				De novo	
30	ISS	7q36 microdeletion syndrome (Kleefstra syndrome 2)	*KMT2C*	deletion				De novo	

Pt. No., participant number; MAF ^a^, minor allele frequency (gnomAD database/Korean Reference Genome Database); in silico analysis ^b^, SIFT/Polyphen2/MutationTaster; FSS, familial short stature; ISS, idiopathic short stature; NAD: nicotinamide adenine dinucleotide; D, deleterious; T, tolerant; PV, pathogenic variant; LP, likely pathogenic; VUS, variant of uncertain significance. Bold indicates a novel mutation.

**Table 3:** Comparison of diagnostic yield using exome sequencing and the genetic results of growth plate dysfunction between SGA SS and non-SGA SS patients.

Patients [Reference] (Number)	SGA [54] (*n* = 176)	SGA Without Imprinting Disorder [55] (*n* = 86)	Non-SGA (ISS) [15] (*n* = 102)	Non-SGA (ISS and FSS) [This Study] (*n* = 41, 36 Families)
Genetic testing	Whole exome sequencing or targeted exome sequencing	Copy number analysis, targeted exome sequencing	Targeted exome sequencing	Targeted exome sequencing
Genetic etiology (CNVs + PV or LP)	74/176 (42%)	13/86 (15.1%)	17/102 (16.7%)	15/36 (41.7%)
Growth plate dysfunction	43/176 (24.4%)	5/86 (5.8%)	17/102 (16.7%)	13/36 (36.1%)
Paracrine signaling	7/176 (4%) *FGFR3, FGFR2, NPR2*	1/86 (1.1%) *NPR2*	8/102 (7.8%) *IHH (4), NPR2 (2), FGFR3 (2)*	3/36 (8%) *NPR2, IHH(VUS), BBS1, TRPS1*
Extracellular matrix	24/176 (13.6%) *ACAN*, various collagens, *FLNB, MATN3* *SHOX* deficiency (7)	*COL2A1(VUS), SHOX (VUS), ACAN (VUS), FGFR3 (VUS)*	4/102 (3.9%) *SHOX, ACAN, ACAN/SHOX* *, COL2A1/NF1 *	4/36 (11%) *COL1A1(VUS, LP), COL2A1(2), MASP1*
Fundamental intracellular/intranuclear processes	12/176 (6.8%) *CDC42, KMT2D, LMNA, NSD1, PTPN11, SRCAP, SON, SOS1, SOX9, TLK2*	4/86 (4.7%) *SMAD4*, *PTPN11*, *CBL*, *MYCN*	4/102 (3.9%) *NF1, PTPN11, BRAF, CBL/SHOX* *	6/36 (17%) *PTPN11(2)*, *SPRED1*, *ADNP*, *CERT1*, *NADSYN1*
Miscellaneous chromosomal aberrations	5/176 (2.8%) del17q24.2 del1p31.1p31.3 del22q11.2 del6q24.3q25.1 dupXq	8/86 (9.3%) del1q21.1q21.2 del22q11.2 dupXp22.33 del19p13.12 dup5q35.2q35.3 (2) del10q26.1q26.3 delXp22.3p11.2/dupXq11.1q28	-	2/36 (6%) dup22q11.2 (*LZTR1*) del7q36 (*KMT2C*)
Pituitary development, GH-IGF-1 or IGF-2 axis, and thyroid axis	14/176 (8%) *LHX4, OTX2, PROKR2, PTCH1, POU1F1*, *GHSR, IGFALS, IGF1R, STAT3, HMGA2, TRHR, THRA*	*GHR(VUS), IGF1R(VUS)*	1/102 (0.9%) *GHSR*	-
Silver–Russell syndrome	12/176 (6.8%) : 11p15, UPD7	Excluded	-	-

SGA, small for gestational age; ISS, idiopathic short stature; FSS, familial short stature; CNVs, copy number variants; PV, pathogenic variant; LP, likely pathogenic variant; VUS, variant of unknown significance; GH, growth hormone; IGF, insulin-like growth factor; UPD, uniparental disomy. * 3 patients had two different gene mutations.

## Data Availability

All data generated or analyzed during this study are included in this article. Further enquiries can be directed to the corresponding author.
